# Implementation and Optimization of Reverse Suspension Structure Design Model Using Deep Learning

**DOI:** 10.1155/2022/7544113

**Published:** 2022-01-30

**Authors:** Xiwen Yu, Kai Wang, Shaoxuan Wang

**Affiliations:** ^1^School of Arts and Media, Hefei Normal University, Hefei, Anhui 230601, China; ^2^School of Science, Anhui Agricultural University, Hefei, Anhui 230036, China; ^3^Shanghai Eigencomm Technologies Ltd., Shanghai 201210, China

## Abstract

The present work aims to improve the design efficiency and optimize the results in the increasingly complex and diversified material design projects to help architects realize the better performance of building structures. According to the characteristics of comprehensive perception and intelligent processing of the Internet of Things, a reverse suspension structure design model is constructed based on the finite element method and simulated annealing algorithm. Besides, deep learning is adopted to train complex functions for performance correction and to optimize the plane structure of shell structure. Moreover, the force is transformed into shape, and the form-finding process is completed to facilitate the operation of designers. Finally, the spatial anchoring ability of the geographic information system is used to match and calculate the relevant characteristics of spatial elements. On this basis, the index construction strategy based on weight distribution is employed to realize the data fusion diagnosis framework and enhance the intelligence of architectural design. The simulation results show that the maximum tensile stress of the physical suspension experiment is 3.71 MPa and the maximum compressive stress is 14.7 MPa. The compressive stress value is much larger than the tensile stress value. The maximum deformation value's difference between the compressive and tensile stress is 0.07 and 0.11, respectively. The error is within the acceptable range, which is similar to the compression state results obtained from the actual suspension physical experiment, indicating that the initial design model of the reverse suspension structure based on deep learning is reliable. In addition, the evolutionary optimization effect analysis results demonstrate that the load of the design structure is relatively uniform, which verifies the feasibility of the algorithm reported here. The research significance of the reverse suspension structure model constructed here is to provide an accurate and feasible design idea for the reverse design of some complex structures in the building suspension. It can also shorten the creation and improvement cycle of this kind of structure and optimize the performance and construction cycle of the building structure.

## 1. Introduction

Now that computer technology develops quickly and simulation software advances continuously, many structural material properties and structure stress can be simulated, analyzed, and optimized. Architects gradually regard structure stress as a new approach to architectural design [1]. This approach enables architects to establish a good relationship between spatial form and structure during structural simulation, calculation, and optimization by analyzing structure stress characteristics in architectural design. Based on this, some specific structural form-finding methods were proposed, such as the force density method [2], dynamic relaxation method [3], a graphical method [4], and thrust grid analysis method [5]. Architectural design is gradually transforming to digital strategies with the continuous improvement in computational efficiency. Digital structural design means that architects perform complex digital simulation, analysis, and optimization via the iterative method of the multipurpose structural design using auxiliary technical tools [6]. Architects can freely optimize the architectural design in more complicated and diverse design projects with algorithms running on computers and expressing various analytic methods based on structure stress. The structural design enables architects to better build structure performance under reasonable economic budget conditions [7]. Therefore, it is of great significance to study the application of computer algorithms in architectural form design.

This design method based on structural mechanics allows architects to simultaneously control, analyze, and optimize the building's material properties and geometric characteristics in a structure model. Moreover, it inspires the architects to rethink the construction feasibility of the design through the systematic analysis of the structure. In this way, new ways for engineers and architects to collaborate are created, conducive to accomplishing more interactive and adaptable structure designs and architectural forms [8]. Architects can design structural forms under reasonable force using structural simulation, analysis, and optimization methods as new design tools [9]. Taking Rhino as the platform, the work focuses on immediate feedback of the calculation process and real-time generation of force-to-shape before applying the methods to actual engineering. The innovations of the present work are as follows: (1) The bidirectional evolutionary structural optimization (BESO) algorithm is adopted to optimize the reverse architectural suspension design model integrating the simulated annealing (SA) algorithm with the finite element analysis (FEA). Besides, the FEA plug-in Karamba and optimization module Galapagos in Rhino simulate the physical test of the shell shape obtained by the model to optimize the shell shape. (2) Topology optimization is performed on the shell structure using the BESO algorithm, obtaining a new shell with a unique shape, reasonable stress, and material saving. At present, the application of geographic information technology to Digital Twins (DTs) still has the following problems. It is difficult to combine it with sensors and other perception technologies at the engine level. The visualization ability is weak, especially in two/three-dimensional integrated application scenarios combined with Building Information Modeling (BIM). Considering the application of geographic information systems (GISs) to DTs, the present work designs and implements a DTs-oriented GIS. This system supports integrated storage, presentation, and analysis of two/three-dimensional data, realizes efficient retrieval of data using a spatial index, achieves the integrated analysis of the perceptional data of sensors via the elements of the spatial correlation characteristics, and optimizes the building function. The present work aims to make effective use of geographic information resources, provide more convenient and efficient DTs application approaches for intelligent buildings, and promote the rapid development of the DTs technology.

The suspension system is a vital component of automobiles. It affects the comfort, safety, and driving control of cars. Therefore, the research and design of parts in automobile suspension systems are fundamental. The steering knuckle and its components are crucial in the automobile suspension system. They bear the weight of the vehicle body and many complex impact loads. Therefore, it is challenging to design and manufacture this kind of automobile chassis part that determines the driving performance of the automobile. China's annual automobile production and sales have surpassed all other countries, including the United States, Germany, and Japan, to become the first in the world. However, China's automotive technology is still relatively backward than traditional automotive powerhouses. Due to automobile technology's late start and development, China's design methods and manufacturing technology are weak. Significantly, the design, research, and application of critical parts in automobile systems are immature, reflecting the vehicle design level of independent research and development. Nevertheless, the design and development of crucial elements need massive financial support, a profound technical foundation, and many high-level professionals. Therefore, utterly independent production in China is not very feasible. Instead, engineers can use the advantages of reverse engineering to study some complex vehicle parts produced by other countries. Then, they can learn the advanced manufacturing technology of some western manufacturing powers by introducing, absorbing, and digesting the technology to improve independent design and manufacturing. The method of reverse engineering based on the existing physical objects has high speed, sound effect, and high application value. It can accurately and efficiently solve the design problems of complex parts in the automotive industry. Meanwhile, the calculation of simulation analysis based on geometric model design combined with computer-aided design can learn advanced technologies. In this paper, the structural design model of reverse suspension in automobile design is transferred to architectural design and optimized.

There are five sections in the present report.  Section 1 is the introduction, which discusses the importance of the practicable algorithm in architectural form design and confirms the research ideas.  Section 2 is a literature review, sketching out related research on architectural form design and application of computer algorithms to force analysis, analyzing the current research status, and clarifying the gaps in the previous study.  Section 3 introduces the research methodology, proposes an architectural design model based on FEA and SA algorithm, and explains optimization details.  Section 4 discusses the research results, analyzes the performance of the model reported here, and compares the reticulated shell before and after optimization.  Section 5 concludes, including actual contributions, limitations, and prospects.

## 2. Related Works

### 2.1. Architectural Form Design

Mechanical analysis of buildings is the most vital link in architectural form design. Sun et al. (2019) proposed the force density method for form-finding analysis of cable net structures. The cable net was regarded as a structural model composed of nodes and rod elements. The intermediate point was the accessible point during form-finding, and the boundary point was the constraint point. A corresponding static balance equation was established for each node in the cable net, and the nonlinear problem was transformed into a linear problem through a preset force density [10]. Boulaud et al. (2020) put forward the dynamic relaxation method, extensively applied in cable net structures. The basic idea of this method was first to discretize the structural system to form a grid. Then, the structural design started an unbalanced force at the initial position. Due to the unbalanced force, the structural system vibrated gradually and point-by-point in space. The structure finally stopped shaking and reached a balanced state until the energy was depleted through the damping term [11]. Rezaiee and Mohammadi (2020) believed that the dynamic relaxation method did not need to assemble and store the global stiffness matrix of the structure. Therefore, the dynamic relaxation method was particularly suitable for dealing with large-scale nonlinear problems, which could find the ideal under the condition that the initially assumed geometry was very unsatisfactory. The initial equilibrium state showed the algorithm's excellent stability and applicability [12]. He et al. (2021) used force density as the ratio of the internal force of the member element to the length of the member in the structure. They solved a set of linear equations and obtained the coordinates of the free nodes in the cable network as the shape of the cable network. The authors found that when the force density method was applied to see the formation of the flexible boundary cable network, an appropriate iterative strategy could make the internal force of the side cable very uniform [13].

### 2.2. Applications of Architectural Design Algorithm

The combination of mechanics and computer algorithms promotes the design method based on the structure stress algorithm. Chieffo et al. (2019) developed an analysis method for arched masonry buildings by illustrating possible structural collapse modes by thrust lines and visualizing the internal forces of the structure by computer simulations [14]. Based on this, they analyzed various architectural forms. They developed corresponding plug-ins which were universally accepted in the project practice of architectural design in different fields, different scales, different functions, and different spatial prototypes [15]. Guo et al. (2020) conducted digital research on shell structure by creating a unique large-span streamline space via shell structure in constructed projects, such as the London Olympic Swimming Pool, Azerbaijan Baku Cultural Center, and Qatar Al Januob Stadium [16]. Houari et al. (2021) reported that the software Karamba 3D studied by Pollinger Guhamman Schneider used FEA to classify systematically and accurately simulate the structural system [17]. Yuan et al. (2021) thought that the topology optimization method had stimulated the development of program plug-ins, which was conducive to the preset of the weight and deformation of the building by designers [18]. Schwartz et al. (2021) believed that algorithms could provide structural data of different materials for reference and comparison. They used structural performance optimization and related knowledge of building materials to turn structural performance analysis tools into an integrated tool for structural morphology generation and optimization [19].

To sum up, in the existing research on architectural design, many scholars use machine learning, computer automation design, and architectural elements to analyze the form and stress of architecture. In architectural design, structural stress analysis refers to the relationship between spatial structure and structural rationality by analyzing structural stress characteristics. This is a continuous calculation, iteration, and optimization process through physical simulation using computer programs. Although there are various structural stress analysis methods, designers are unfamiliar with them and have not been widely used in practical engineering. Therefore, this paper summarizes four structural stress analysis methods using the interactive structural stress analysis method as the mainline. Taking an actual project as an example, these methods are applied to building structure optimization and shape design. The shape results are analyzed by the finite element method to verify its reliability and superiority.

## 3. Research Methodology

### 3.1. Overview and Analysis of the Algorithm Procedure

**Figure 1:**
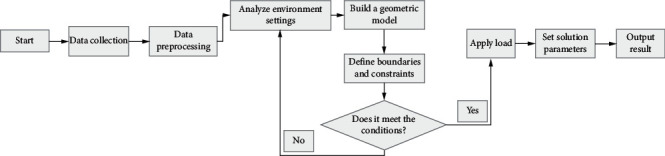
Analysis process of stress strength by FEA.

The FEA method is a critical tool for scientific research. It can solve various complex mathematical and physical problems and solve complicated engineering problems. The rationale of FEA is to separate the model with arbitrary shape from the specific grid and obtain the solution of the whole domain by solving the subdomain [20]. Elasticity is the mechanical basis of the FEA of solid structures. The equation solving adopts the principle of weighted residual method or extreme functional value, which is realized by discrete numerical technology and takes the FEA software as the technical carrier. Due to extensive calculation, it is essential to deal with practical problems on the computer hardware platform [21]. When a deformable body is subjected to external action, the description of the force and material characteristics are indirect, and new variables need to be defined. When the material is determined, the primary mechanical variables include the displacement component (describing the position of the object after deformation), strain component (representing the deformation degree of the thing), and stress component (describing the stress state of the object).  Figure 1 illustrates the analysis process of the stress intensity of building structures by the FEA algorithm here.

**Figure 2:**
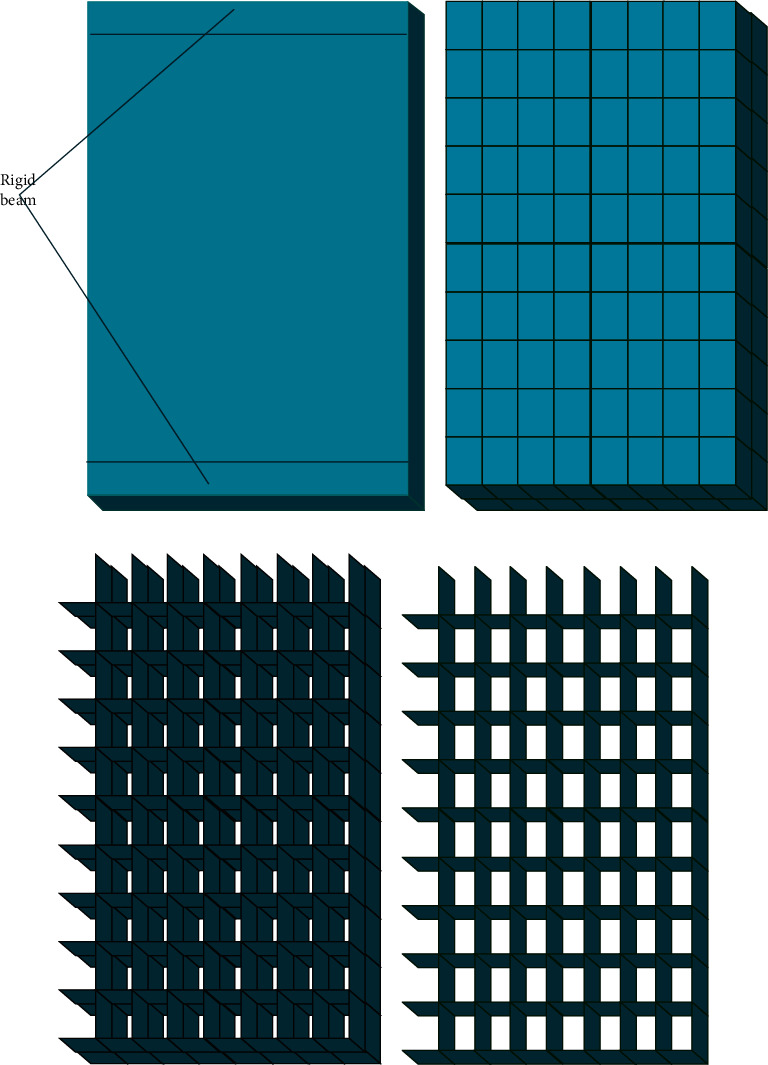
Wall model for analyzing the stress on wall steel plates. (a) Wall structure. (b) Steel plate and concrete structure. (c) Steel plate and section steel structure. (d) Single-layer structure.

As shown in Figure 1, FEA can obtain a specific object's displacement, strain, and stress by solving equilibrium equations, geometric equations, and physical equations. Boundary conditions refer to the equation system's requirements on the moving boundary. There are two boundary conditions for general mechanical problems: displacement and force boundary conditions. By limiting boundary conditions, these three variables can be solved faster. Taking the stress on wall steel plates in architectural design as an example to model and analyze the change of building stress, the wall model is shown in Figure 2.

**Figure 3:**
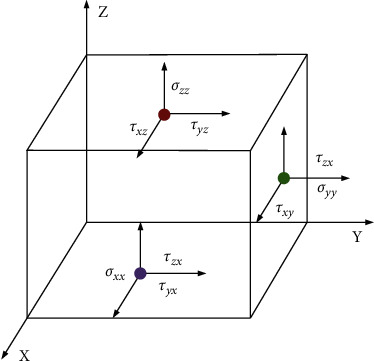
Stress components in space.

The stress component is demonstrated in Figure 3.

The equilibrium equations can be expressed as

(1) $$\begin{array}{c} \frac{\partial \sigma_{xx}}{\partial_{x}}+\frac{\partial \tau_{xy}}{\partial_{y}}+\frac{\partial \tau_{zx}}{\partial_{z}}+\bar{X}=0,\\ \frac{\partial \tau_{xy}}{\partial_{x}}+\frac{\partial \sigma_{yy}}{\partial_{y}}+\frac{\partial \tau_{zy}}{\partial_{z}}+\bar{Y}=0,\\ \frac{\partial \tau_{zx}}{\partial_{x}}+\frac{\partial \tau_{yz}}{\partial_{y}}+\frac{\partial \sigma_{zz}}{\partial_{z}}+\bar{Z}=0. \end{array}$$

The geometric equations can be written as

(2) $$\begin{array}{c} \varepsilon_{xx}=\frac{\partial_{u}}{\partial_{x}},\\ \varepsilon_{yy}=\frac{\partial_{v}}{\partial_{y}},\\ \varepsilon_{zz}=\frac{\partial_{w}}{\partial_{z}},\\ \gamma_{xy}=\frac{\partial_{v}}{\partial_{x}}+\frac{\partial_{u}}{\partial_{y}},\\ \gamma_{yz}=\frac{\partial_{w}}{\partial_{y}}+\frac{\partial_{v}}{\partial_{z}},\\ \gamma_{zx}=\frac{\partial_{w}}{\partial_{x}}+\frac{\partial_{u}}{\partial_{z}}. \end{array}$$

The physics equations can be presented as

(3) $$\begin{array}{c} \varepsilon_{xx}=\frac{1}{E}\left[\sigma_{xx}-\mu \left(\sigma_{yy}+\sigma_{zz}\right)\right],\\ \varepsilon_{yy}=\frac{1}{E}\left[\sigma_{yy}-\mu \left(\sigma_{xx}+\sigma_{zz}\right)\right],\\ \varepsilon_{zz}=\frac{1}{E}\left[\sigma_{zz}-\mu \left(\sigma_{xx}+\sigma_{yy}\right)\right]. \end{array}$$

The displacement boundary conditions are determined according to

(4) $$\begin{array}{c} u\left(x,y,z\right)|x=x_{0},y=y_{0},z=z_{0}=\bar{u},\\ v\left(x,y,z\right)|x=x_{0},y=y_{0},z=z_{0}=\bar{v},\\ w\left(x,y,z\right)|x=x_{0},y=y_{0},z=z_{0}=\bar{w}. \end{array}$$

**Figure 4:**
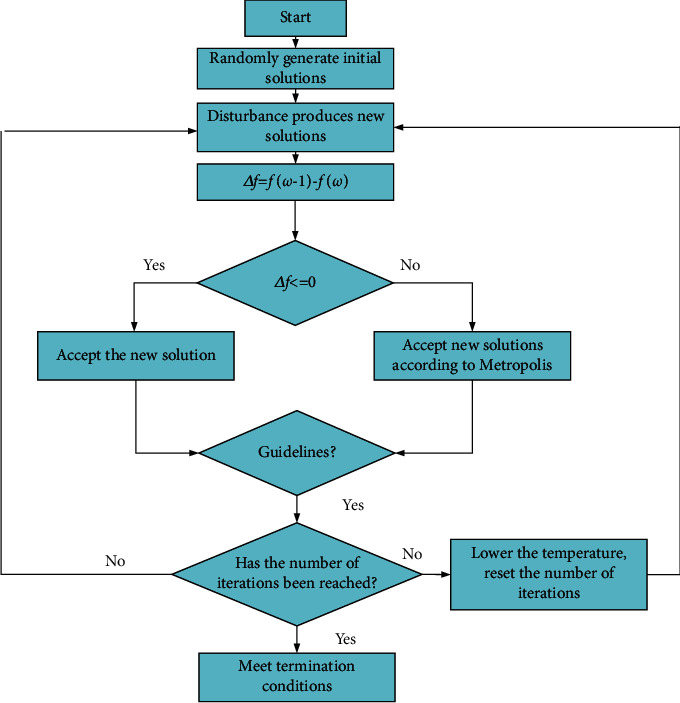
Basic procedures of SA algorithm.

**Figure 5:**
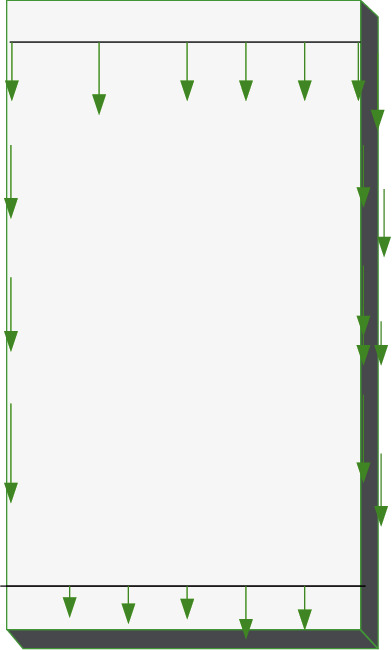
Structural loads with muscular stress (the arrow in the figure indicates the stress direction).

The force boundary conditions can be defined as

(5) $$\begin{array}{c} n_{x}\sigma_{xx}\left(x_{0},y_{0},z_{0}\right)+n_{y}\sigma_{xy}\left(x_{0},y_{0},z_{0}\right)+n_{z}\tau_{xz}\left(x_{0},y_{0},z_{0}\right)=\bar{p_{x}},\\ n_{x}\tau_{xy}\left(x_{0},y_{0},z_{0}\right)+n_{y}\sigma_{yy}\left(x_{0},y_{0},z_{0}\right)+n_{z}\tau_{xz}\left(x_{0},y_{0},z_{0}\right)=\bar{p_{y}},\\ n_{x}\tau_{xz}\left(x_{0},y_{0},z_{0}\right)+n_{y}\tau_{xy}\left(x_{0},y_{0},z_{0}\right)+n_{z}\sigma_{zz}\left(x_{0},y_{0},z_{0}\right)=\bar{p_{z}}, \end{array}$$

where the meaning of ∂_*x*_, ∂_*y*_, ∂_*z*_, *σ*_*xx*_, *τ*_*xy*_, *τ*_*zx*_, *τ*_*xy*_, *σ*_*yy*_, *τ*_*zy*_, *τ*_*zx*_, *τ*_*yz*_, and *σ*_zz_ are shown in the legend in Figure 3; *x*_*0*_, *y*_*0*_, and *z*_*0*_ denote the origin of coordinates; *E*, *μ*, and *G* are the elastic modulus, Poisson's ratio, and shear modulus of the material. Besides, the two subscripts in stress *s* and *τ* represent the direction of the stress and the normal direction of the stress acting surface, respectively, and the same is true for strain *ε* and *γ*. In addition, $\bar{x},\bar{y}$, and $\bar{z}$ refer to the physical strength components in different directions, *x*_*0*_, *y*_*0*_, and *z*_*0*_ represent geometric boundary coordinates, and *n*_*x*_, *n*_*y*_, and *n*_*z*_ denote the cosine of the normal outside the boundary. Besides, $\bar{u},\bar{v}$, and $\bar{w}$ stand for the displacement in the corresponding direction, and $\bar{p_{x}},\bar{p_{y}}$, and $\bar{p_{z}}$ indicate the distributed force of the boundary in the corresponding direction. In the boundary conditions of FEA, it is assumed that sufficient structural measures are taken to firmly bond steel plates, section steel, and concrete. Therefore, the steel plate, section steel, and concrete models can be tied together by constraints during simulation. The rebar is embedded inside the concrete body. In the model, the bottom of the shear wall is tied with the loading beam and fully fixed with the ground. In other words, the bottom constraint condition is end fixing, and there is no translational and rotational displacement, as presented in Figure 4. The top of the shear wall is also tied to the loading beam. The loading beam is a rigid beam of steel, three orders of magnitude increase the elastic modulus, and no other characteristics are defined. Under these settings, the load can be uniformly transmitted to the shear wall component.  Figure 5 reveals the stress intensity and structural load.

SA algorithm is a heuristic random optimization algorithm that selects the state with the smaller target value in the adjacent area with a specific probability. The internal particles tend to be turbulent and irregularly arranged when the temperature of the solid increases. On the contrary, they slowly solidify stably as the temperature gradually decreases, and the reliable gradually attains a stable state accordingly. During annealing, the energy state of the object gradually decreases with the temperature. When the energy state reaches the minimum, the thing assumes the shape of crystalline [22]. The SA algorithm repeats the iterative solution according to the probabilistic jumping property during heating and cooling and finally obtains the optimal global solution to the problem. Implementation procedures for the algorithm are as follows. The initial temperature is set to *T*_*0*_, which is high enough. Suppose that *T* = *T*_*0*_ to calculate initial solution *S*_*1*_, and the iteration times at every time *T* are determined. Repeat the following steps regarding the current temperature *T* and *K* = 1, 2,…, *L*. First, a new solution *S*_*2*_ is generated for the random disturbance of the current solution. Second, the increment of *S*_*2*_ is expressed as *df* = *f*(*S*_*2*_) – *f*(*S*_*1*_). If *df* < *0*, *S*_*2*_ will be accepted as the current solution. Otherwise, the acceptance probability of *S*_*2*_ is denoted as exp(*-df*/*T*). Third, a random number rand with uniform distribution is randomly generated within the interval of (0, 1). If e*xp*(*-df*/*T*) > rand, *S*_*2*_ is taken as a new current solution, *S*_*1*_*= S*_*2*_. Otherwise, the current solution *S*_*1*_ is retained. If the termination condition is met, the current solution *S*_*1*_ is regarded as the optimal solution, ending the program [23]. As a general random search algorithm, the SA algorithm has been widely used in VLSI design, image recognition, and neural network computer research. It can jump out of the optimal local trap. Even if the system falls into the local optimization in the Boltzmann machine, the algorithm can jump out after some time. The system will eventually converge to the direction of global optimization. It is often used in other combinatorial optimization problems. Multitudes of simulation experiments show that the SA algorithm quickly produces satisfactory approximate optimal solutions. SA algorithm is primarily used under challenging problems to obtain specific solutions accurately. Through multiple iterations, it can continuously approach the optimal solution. It has the advantages of the simple calculation process, universality, strong robustness, applicability to parallel processing, and feasibility to complex nonlinear optimization problems.  Figure 5 displays the general solution process of the SA algorithm.

### 3.2. Model Design and Construction

**Figure 6:**
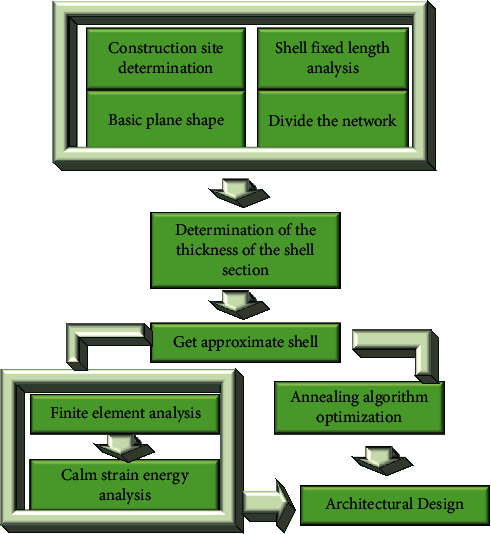
Flowchart of shell shape determination and optimization design.

This section takes the concrete shell structure as an example. According to the above shell form-finding idea [24], the FEA method and SA algorithm are combined, and the Karamba and Galapagos plug-ins of the parametric platform Grasshopper are used to optimize the shell structure. The specific framework is shown in Figure 6. FEA is performed on the special-shaped concrete roof shell, and the parameter values are determined by referring to the relevant regulations of the Technical Specification for Spatial Grid Structure [25]. The length of the building site is 74 m, the width is 40 m, and the structural height is set at 11 m. Besides, the self-weight of the structure and the live load of the roof is 0.5 kN/m^2^, the load combination equals 1.0 self-weight + 1.0 live load, and the concrete strength grade of the shell is C40.

Here is the design process: ① determine the size of the site and the length of the fixed end to obtain a plan shape; ② determine the height of the target shell, apply the vertical self-weight load, and use the extensive deformation analysis to simulate the reverse physical suspension of the plane; ③ obtain a reverse physical suspension model, a shell under pure pressure; ④ determine structural parameters of the body, such as load, material, and boundary conditions. Determine the initial value of section thickness, conduct FEA on the obtained shell model, and solve its average strain energy; ⑤ take the interception proportion and section thickness as independent variables, and the average strain energy as the dependent variable. Input the variables into the SA algorithm for calculation. The algorithm can solve the minimum average strain energy within the value range of independent variables until the optimal solution is obtained [26].

### 3.3. BESO

As the FEA method grows and is widespread, structural topology optimization has also been applied to structural design in various fields. Structural topology optimization can produce optimal structural shapes according to different constraints and objective functions, so it is generally used to study continuum structures. The BESO algorithm is a mature topology optimization method of continuum structures [27]. Structural topology optimization aims to obtain the best structural performance by searching for the structure's optimal topological form, shape, and size under particular constraints [28]. BESO is utilized to optimize the structural model, which can be expressed as

(6) $$\begin{array}{c} \text{find:}\;X={\left\{x_{1},x_{2},\ldots ,x_{n}\right\}}^{T}\in \Omega , \end{array}$$

(7) $$\begin{array}{c} \text{minimize:}\;C=\frac{1}{2}F^{T}U, \end{array}$$

(8) $$\begin{array}{c} \text{subject to}:\;V*-\sum_{i=1}^{n}V_{i}x_{i}=0, \end{array}$$

(9) $$\begin{array}{c} F=KU, \end{array}$$

(10) $$\begin{array}{c} x_{i}=\left\{x_{\min},1\right\},\;\left\{i=1,2,\ldots ,n\right\}. \end{array}$$

In equations (6)–(10), *x*_*i*_ refers to the design variable in structural optimization, namely, the density value of the unit, and *n* describes the total number of units in the structural design area, while *V∗* and *V*_*i*_ are the target volume and the volume of each unit, respectively. Besides, *F* denotes the load vector, *U* refers to the displacement vector, and *C* signifies the strain energy of the structure. According to the above equations, the relationship between the elements in the continuum structure and the strain energy in topology optimization can be derived, as shown in

(11) $$\begin{array}{c} C=\frac{1}{2}F^{T}U,\\ =\frac{1}{2}U^{T}KU\\ =\frac{1}{2}\sum_{i=1}^{n}u_{i}^{T}k_{i}u_{i}. \end{array}$$

In equation (11), *k*_*i*_ refers to the element stiffness matrix, and *u*_*i*_ represents the column vector of element displacement. The original evolutionary structural optimization introduces a material interpolation form with a penalty factor, which sets the elastic modulus of the material to

(12) $$\begin{array}{c} E\left(x_{i}\right)=E_{0}x_{i}^{p}. \end{array}$$

In equation (12), *x*_*i*_^*p*^ represents the unit material density, *E*_0_ indicates the elastic modulus of the element material, and *p* refers to the penalty factor. The material interpolation form with a penalty factor can polarize the unit material. The penalty factor can prevent the appearance of the checkerboard form by eliminating the intermediate unit. The average strain energy expression of the structure with the penalty factor is illustrated in

(13) $$\begin{array}{c} C\left(x_{i}\right)=\frac{1}{2}\sum_{i=1}^{n}x_{i}^{p}u_{i}^{T}k_{0}u_{i}. \end{array}$$

In equation (13), *k*_0_ refers to the stiffness matrix of the solid element. According to equation (13), an optimization model in material interpolation form with a penalty factor can be established, taking the strain energy of the structure as the optimization goal and the volume of structures as the constraint. The structural optimization model is described as the following equations:

(14) $$\begin{array}{c} \text{find}:\;X={\left\{x_{1},x_{2},\ldots ,x_{n}\right\}}^{T}\in \Omega ,\\ \text{minimize:}\;C=\frac{1}{2}F^{T}U,\\ =\frac{1}{2}U^{T}KU\\ \text{subject to}:\;V*-\sum_{i=1}^{n}V_{i}x_{i}=0. \end{array}$$

At the beginning of optimization, the units must be sorted in descending order of sensitivity. The unit with lower sensitivity should be changed into empty units according to the preset unit deletion ratio until the structure meets the convergence and volume constraints to obtain an optimal topology. The sensitivity value associated with whether the unit is removed or left affects the final design by topology optimization, a critical factor in the entire process. The sensitivity value of the *i*-th unit is the partial derivative of the objective function to the *i*-th design variable, which is the unit density, as described in

(15) $$\begin{array}{c} \frac{\partial C}{\partial x_{i}}=-\frac{p}{2}x_{i}^{p-1}u_{i}^{T}k_{o}u_{i}. \end{array}$$

In BESO, the value of the design variable *x*_*i*_^*p*−1^ is limited to 1 or *x*_min_. Then, the sensitivity of the unit can be expressed as

(16) $$\begin{array}{c} \alpha_{i}=-\frac{1}{p}\frac{\partial C}{\partial x_{i}},\\ =\left\{\begin{array}{c} \frac{u_{i}^{T}k_{o}u_{i}x_{i}}{2}\\ \frac{x_{i}}{2}x_{i}^{p-1}u_{i}^{T}k_{o}u_{i}, \end{array}\right.. \end{array}$$

Equation (16) can be simplified as

(17) $$\begin{array}{c} \alpha_{i}=-\frac{1}{p}\frac{\partial C}{\partial x_{i}},\\ =\left\{\begin{array}{cc} \frac{u_{i}^{T}k_{o}u_{i}}{2}, & x_{i}=1\\ 0, & x_{i}=x_{\min}. \end{array}\right. \end{array}$$

**Figure 7:**
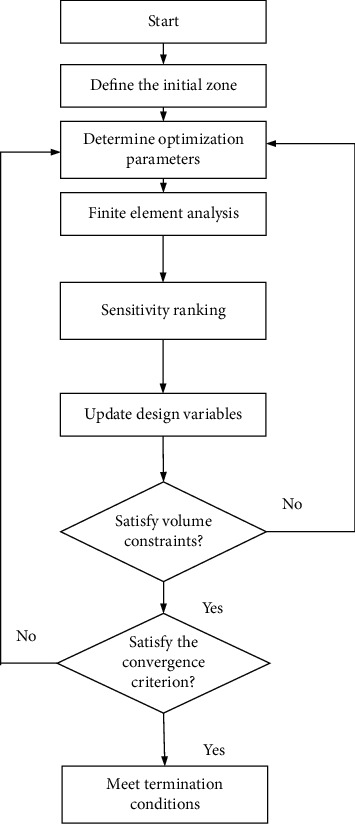
Optimized procedure of BESO.

Figure 7 illustrates the implementation process of the bidirectional evolutionary optimization algorithm. Firstly, the optimal region of the structure and the given boundary conditions are determined. Secondly, the initial parameters of optimization are defined. Thirdly, the compliance of the frame and the sensitivity of each element are calculated. Fourthly, the element sensitivity is updated. Fifthly, the sensitivity value of the unit is arranged according to the size to determine the sensitivity threshold of the unit optimization. Sixthly, it is essential to determine whether the target volume has been achieved.

### 3.4. Application of DTs to Architectural Function Design

**Figure 8:**
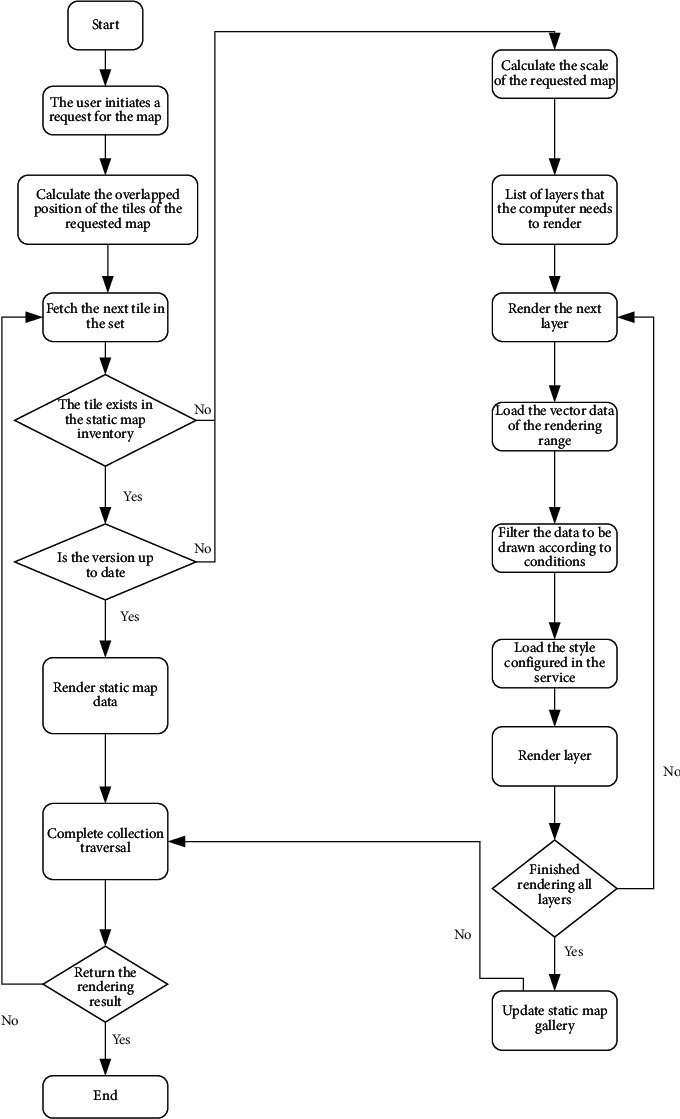
Rendering process of two-dimensional maps.

In the application scenarios facing DTs, business data may cover all aspects of urban governance, and the boundary between static and dynamic maps is even more blurred. Therefore, a fusion way of static maps and dynamic maps is designed here, and the state flag byte is added.  Figure 8 reveals the two-dimensional map rendering process of the system.

**Figure 9:**
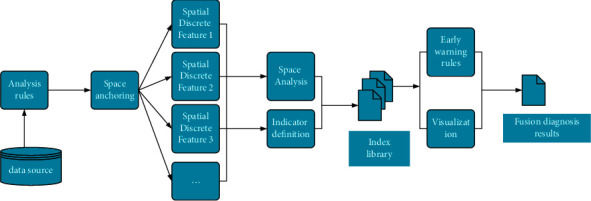
Process of the spatial fusion diagnosis.

When receiving the user's map request, the system first calculates the tile location set in the requested map range, then traverses the tile location in the collection, and finds the tile-map data of the corresponding site from the static map library. If the state in the library where the data exists is the latest, the tile is directly rendered to the resulting map; otherwise, the system requests the scale of the map and combines it with the map service configuration to get the set of vector layers that the tile needs to render. Then, the system iterates through all vector layers, retrieves vector data from the database against the spatial index, and renders tiles according to the style defined in the service configuration. Next, the tiles are generated into the resulting image and updated into the static map library simultaneously, and the rendered image is returned to the user. When the business system changes the map data and identifies the tiles involved in the data as outdated, the data update will be triggered when another user requests the area map. The data diagnosis of the system is based on the analysis model of the system.  Figure 9 illustrates the process of the spatial fusion diagnosis based on DTs.

### 3.5. Project Overview and Simulation

The analysis object is the V-shaped light well structure design for the Dunhuang Mogao Grottoes Tourist Service Center. Here, Rhino software is used as an analysis tool to optimize the design of the light well structure, achieve reasonable structural stress and diverse shapes, and save materials. The construction plan size of the project site is about 94 *m* × 215 m, the construction area is 11825 m^2^, and the maximum height above the ground is 15.8 m. The V-shaped light well is located between the souvenir sales office and the restaurant. Furthermore, the projection size of the V-shaped light well is about 26 *m* × 19 m, the maximum height is 9 m, and it adopts a grid structure of beams and columns. The structural safety level of the project is Class II, the seismic fortification category is Class B, the foundation design is Class B, and the seismic fortification intensity is 7°. The concrete strength grade is C40, the tensile strength is 2.39 Mpa, and the compressive strength is 26.8 Mpa [29].

**Figure 10:**
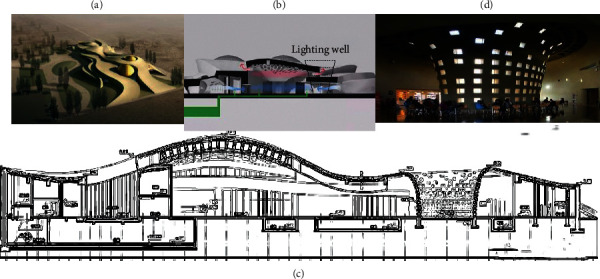
Structure of the light well. (a) Project structure diagram. (b) Location of light well. (c) Section view of the light well. (d) Site picture of light well.

Then, the initial plane projection is obtained according to the plan view and the projection size of the light well. Based on the cross-sectional view of the light well, the overall building structure is uneven. The left and right heights of the light well are 7.542 m and 8.378 m, respectively. Therefore, the initial plane's height settings on the left and right sides are consistent with the actual structure. Since the maximum height of the light well is 9 m, the value in this item is determined to be 9 m. “Angle Tolerance” is set to 7.5, which is the maximum deviation between the corresponding edges of the graph and the force graph. The state of horizontal equilibrium allows a deviation range of 5–10°. The material of the shell is C40 concrete, and the sensitivity filter radius should not be smaller than the mesh size, which is set to 2 m [30]. Here, StructureFIT is adopted to realize the shape optimization of the human-computer interactive plane truss. The optimized plane truss is analyzed by FEA and compared with the original structural design. The FEA plug-in Karamba and the optimization module Galapagos in Rhino are employed to simulate the physical test of the shell shape obtained by the reverse suspension model for shape optimization. Ansys Workbench is used as the FEA software.  Figure 10 shows the fundamental structure of the light well. The performance of geographic information and building application system based on DTs is tested. The test environment is published with a single node of a virtual server, and the configuration is summarized in Table 1.

As shown in Table 1, the test scope includes the operation response speed of the three core functions, namely, dynamic map rendering, concurrent map rendering, and spatial data query. Each test result is the mean value of 10 repetitions.

## 4. Result Analysis

### 4.1. Annealing Finite Element Simulation Results

**Figure 11:**
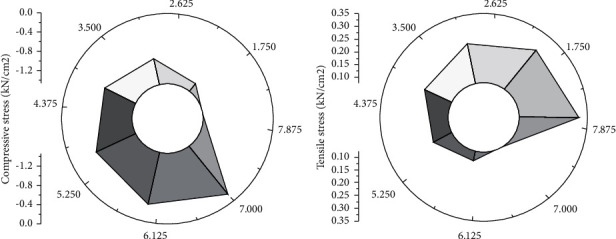
Force analysis results by the suspension simulation model. (a) Compressive stress results. (b) Tensile stress result.

Figure 11 reveals the stress analysis results of the suspension model, where Figure 11(a) shows the compressive stress results and Figure 11(b) illustrates the tensile stress results.

Using FEA and the SA algorithm can ensure a smooth development of suspension physics experiments. Most areas of the structure are red, indicating extensive compression. The maximum tensile stress is 3.71 Mpa, while the maximum compressive stress is 14.7 Mpa. Compressive stress is much larger than the tensile stress value, similar to the compression state obtained by the actual suspension physics experiment.

**Figure 12:**
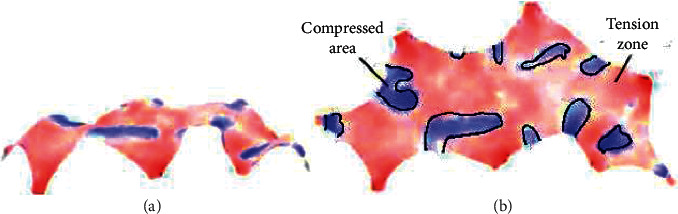
Average strain energy of the optimized structure. (a) Stress map before parameter optimization. (b) Stress map after parameter optimization.

Figure 12 shows the average strain energy of the structure after optimizing the parameters.

The ShellView component is used to visualize the calculation results, and the structural stress is checked. In Figure 12, the red part is the compressed area, and the blue part is the tensioned area. From Figure 12, most areas of the structure are red. Due to the vertical downward external load, the solid shell produces partial tension. The scope of the red (compression) area of the structure is much larger than that of the blue (tension) region, which proves that the shell shape meets the user's expectation of the shell [31].

**Figure 13:**
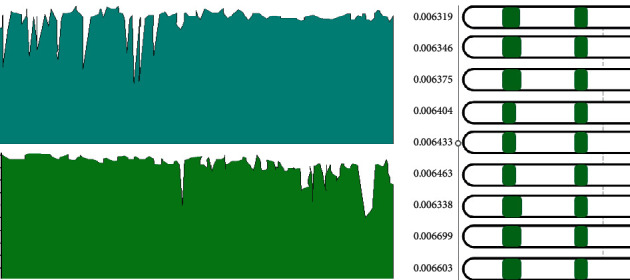
Shell parameter structure optimization.

As shown in Figure 13, two independent variables (the fixed end length and the shell section thickness) and a dependent variable (the average strain energy) are input into the Galapagos arithmetic unit. The SA algorithm can find the optimal solution with the most negligible average strain energy. The initial temperature is set to 100°C, the cooling degree is 95%, and the jumping probability is 25%.

### 4.2. Optimization and Visual Analysis

**Figure 14:**
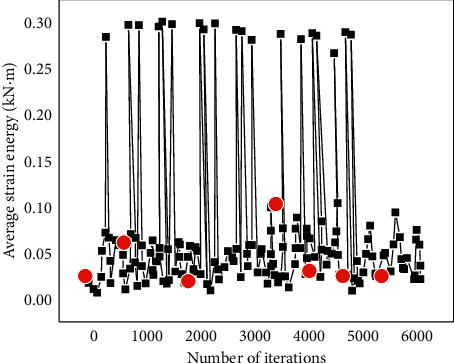
Changing process of average strain energy during optimization.

Figure 14 displays the model's average strain energy variation process during the optimization process.

**Figure 15:**
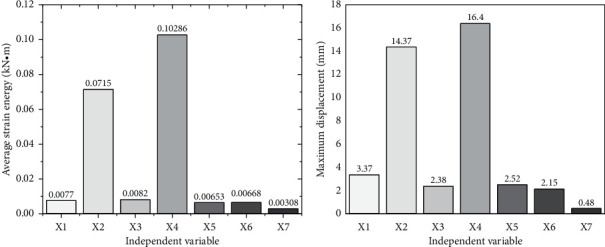
Visualized comparison results of the optimization process. (a) Average strain energy change. (b) Maximum displacement change.

As shown in Figure 14, based on the Rhino platform, Galapagos can visualize the optimization process and results in real-time to record the change of the average strain energy in the optimization process. Therefore, seven red dots in Figure 14 are randomly selected to compare the structural optimization process and further explore the change of the average strain energy in the model optimization process. The research results are presented in Figure 15.

X1 ∼ X7 in Figure 13 are the red dots in Figure 14.  Figure 15(a) indicates the analysis results of average strain energy of 7 different positions, and Figure 15(b) represents the maximum displacement of seven places. When the length ratio in X7 is 0.056, and the shell thickness is 36 cm, the average strain energy of the shell is the lowest, reaching 0.00308 kN/m, and the maximum displacement is 0.48 cm. Compared with the initial stage, the average strain energy is reduced by 60.4%, and the deformation is reduced by 85.8%. The tensile strength of concrete materials is much lower than the compressive strength. Therefore, avoiding structural tension is also crucial for designing concrete shell structures. According to the figures reported here related to the shell analysis, the red area of the optimized shell, that is, the compression area, increases significantly, which is considerably larger than the blue (tension) area.

**Figure 16:**
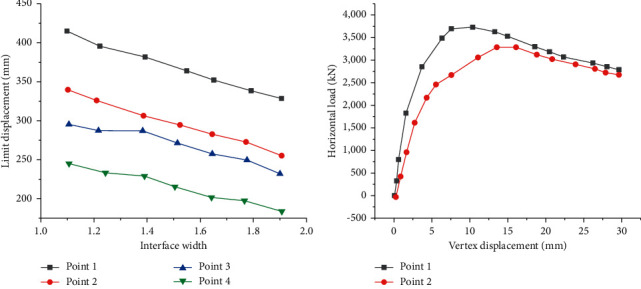
Relationship between the height-width ratio and bearing capacity of the building structure: (a) the relationship between height-width ratio and bearing load; (b) the relationship between load and displacement.

Then, components with different height-width ratios under the same section size are selected for FEA to obtain each component's limit displacement curves and vertex displacement curves and discuss the relationship between the height-width ratio and bearing capacity. The statistical results are shown in Figure 16.

Through Figure 16, under the same section size, the shear wall's initial stiffness and bearing capacity tend to decrease with the increase in height-width ratio. Besides, under the same section size, the ultimate displacement of a wall with a section height-width ratio of 2.0 is greater than that of a wall with a height-width ratio of 1.5. The limit displacement values of Point 1 and Point 2 are 36.9% and 47.6% higher than those of Point 4, respectively. Nevertheless, this does not mean that the deformation capacity of the wall with a larger height-width ratio is better. The ultimate interlayer displacement angle is another index to evaluate the deformation capacity of the shear wall. It refers to the percentage of maximum displacement to floor height. The maximum interlayer displacement angles of Points 1–4 are 4.39 mm/m, 4.51 mm/m, 3.94 mm/m, and 4.36 mm/m, respectively. There is no significant increase between the ultimate interlayer displacement angles of walls under two height-width ratios.

**Figure 17:**
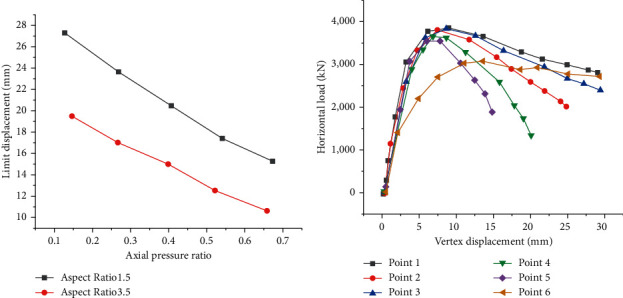
Relationship between axial compression ratio and a bearing load of the building structure: (a) relationship between axial compression ratio and limited bearing load; (b) the relationship between load and displacement under different axial compression ratios.

Another factor dramatically impacting the building strength is the axial compression ratio. To quantitatively analyze the improvement of the horizontal bearing capacity of the wall by the axial pressure, FEA is carried out for the components with different axial compression ratios under the condition of the same section size.  Figure 17 provides the relationship between the axial compression ratio and the bearing load of the building structure.

From Figure 17, the axial compression ratio greatly influences the bearing capacity of steel plate-concrete composite shear wall. When the axial compression ratio is small, the axial pressure can improve the horizontal bearing capacity of the shear wall. Moreover, with the increase in the axial compression ratio, the horizontal bearing capacity of the shear wall increases gradually. After reaching a specific limit, if the axial compression ratio continues to grow, the horizontal bearing capacity of the wall begins to decrease.

**Figure 18:**
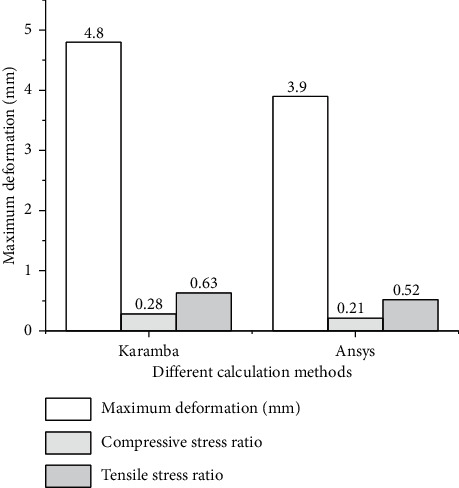
Indicator performance and error of different calculation methods.

According to Figure 18, the calculation results by Ansys Workbench are compared with those of Karamba. The comparative analysis demonstrates that there is no apparent difference between the two. Therefore, based on the Karmaba and Galapagos plug-ins, combining FEA and SA algorithm is feasible for the initial structural design.

As shown in Figure 18, calculation results of the FEA algorithm are compared with those of Karamba. From the perspective of the maximum deformation value, the result of FEA is 3.9 mm, while the value of Karamba is 4.8 mm, with a difference of 0.9 mm. Besides, the difference between compressive stress and tensile stress is 0.07 and 0.11. From the comparative values of the two groups of methods, the error is within the acceptable range, demonstrating that the initial structural design combining FEA and SA is reliable.

### 4.3. Evolutionary Optimization Effect Analysis

**Figure 19:**
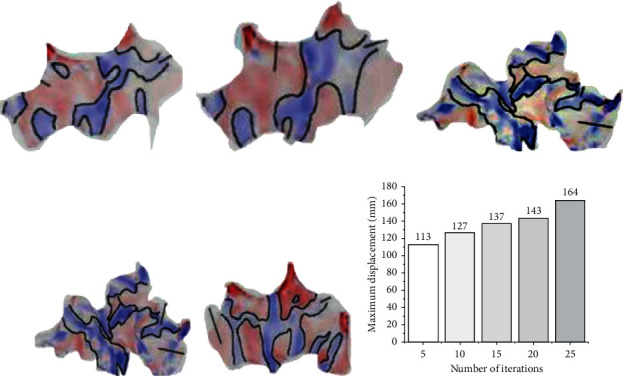
Evolutionary optimization effect analysis.

Figure 19(a) shows the stress indexes of the model under different evolution indexes, and Figure 17(b) presents the performance results of the model under different iteration times.

In Figure 19(a), the deeper the red, the more severe the pressure; the darker the blue, the lighter the pressure. The proportions of the red and blue areas in the structure are relatively close, indicating that the designed structural load is fairly uniform. From Figure 19(b), the maximum displacement of the optimized format is less than 1/200 of the short span of 40 m. This meets the structural design requirements and verifies the reliability of the BESO algorithm. With the gradual growth of the number of iterations, the number of materials is decreasing, the structural form is changing, the structure presents an irregular shape, and the system's novelty is increased.

### 4.4. Test Results of the Core Performance of the DTs Building Geographic Information System

**Figure 20:**
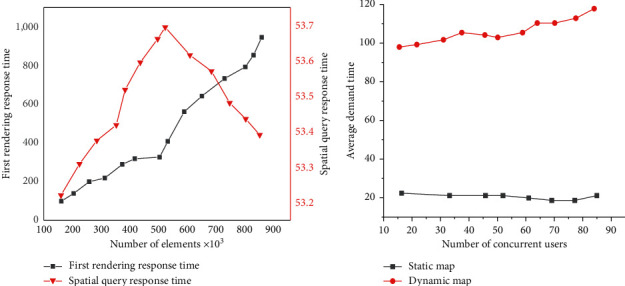
Test results of the response time of the DTs building geographic information system: (a) rendering and query response time; (b) concurrent user response time.

The core performance of the DTs building geographic information system is evaluated by the response time, and the test results are shown in Figure 20.

According to Figure 20, the dynamic map is updated to a static map after the first drawing due to the system's architecture design. First, the first drawing efficiency of a single user accessing the active map is tested. The system rendering time increases with the increase of elements from the data, and the linearity is good. According to the relationship between the spatial query response time of the system and the amount of layer data, the random irregular polygon is generated by the algorithm for spatial data query. Moreover, the query efficiency of the system is not related to the total amount of layer data, which is also consistent with the principle of a spatial index designed by the system. Furthermore, when many users are concurrent, with the increase of simultaneous users, the mapping response time of static maps is almost unchanged, related to the use of LAN in the test environment. On the contrary, the mapping response time of dynamic maps increases linearly with the increase in users. Still, the growth is relatively slow, which shows that the system's concurrency is satisfying.

To sum up, based on architectural design research and related algorithms, FEA and SA algorithms are integrated to build a reverse architectural suspension design model. Besides, the BESO algorithm and software are employed to optimize the shell of the plane structure obtained by the model. Moreover, the example data provides convenience for designers' operations. The strength design method of the building structure is optimized from two aspects of stress visualization and quantification, which offers reference and ideas for relevant research. Some scholars studied the effectiveness of a nonsmooth semiactive control algorithm to suppress the vibration performance of building structures under seismic waves. According to Lyapunov stability theory, it has been proved that the nonsmooth semiactive control algorithm can realize the finite-time stability of vibration relative to the isolation layer of the building structure. In addition, Fairuz et al. (2020) compared and analyzed the vibration conditions of passive control, semiactive control, and nonsmooth semiactive control through the numerical simulation of two buildings with different parameters under seismic wave input [32]. Through simulation, they found that the nonsmooth semiactive control algorithm had good robustness and effectiveness in restraining the influence of earthquakes on the structure. Compared with the above research, the present work introduces the BESO algorithm based on stress monitoring and strength verification, making the strength design of the building structure more flexible and novel. Besides, employing DTs technology makes the architectural design more intelligent.

## 5. Conclusions

FEA and SA algorithms are combined to establish the structural design model based on the form-find design and related algorithms. The plane structure design of the shell structure is optimized and analyzed by the corresponding software. In addition, the BESO algorithm refines the form-finding process of transforming force into shape and visualizing topology. Finally, DTs technology is applied to construct the DTs-oriented building GIS. The spatial anchoring ability of GIS is used to match and calculate the relevant characteristics of spatial elements. On this basis, the index construction strategy based on weight distribution is adopted to realize the data fusion diagnosis framework based on spatial correlation analysis and improve the intelligence of architectural design.

However, there are still many deficiencies in this paper. Firstly, only one example is selected to verify each structural form-finding method. Further research will select examples of different shapes and working conditions to optimize and improve the reliability of the form-finding way. In addition, many problems encountered in engineering practice are still solved by experience, and a lot of work needs to be done to find out the quantitative law. For example, wind load, snow load, and rain load should be considered for the load effect of membrane structure. However, due to the various surface forms of membrane structure, the shape coefficient of wind load is not specified in the specification. Determining the shape coefficient of snow and rain loads still needs to be further studied and standardized. Secondly, the stress of shell structure is complex. Accordingly, it is necessary to carry out experimental research on the optimized shell structure to understand the optimized structural performance more accurately and verify the reliability of the structural form-finding method through the actual test results.

## Figures and Tables

**Table 1:** Test environment configuration.

Project	Configuration
CPU	Xeon E5-2620@ 2.10 Hz (2 processor)
RAM	16 GB DDR4 2400 MHz
Disk capacity	100 GB
Operating system	Windows Server 2008 R2 Standard
Database	SQL Server 2014
Network	Gigabit routing, local area network

## Data Availability

The data used to support the findings of this study are included within the article.
